# Focusing on mitochondria in the brain: from biology to therapeutics

**DOI:** 10.1186/s40035-024-00409-w

**Published:** 2024-04-17

**Authors:** Nanshan Song, Shuyuan Mei, Xiangxu Wang, Gang Hu, Ming Lu

**Affiliations:** 1 Department of Pharmacology, School of Medicine & Holistic Integrative Medicine, Nanjing University of Chinese Medicine, Nanjing, 210023 China; 2 The First Clinical Medical College, Nanjing Medical University, Nanjing, 211166 China; 3 Jiangsu Key Laboratory of Neurodegeneration, Department of Pharmacology, Neuroprotective Drug Discovery Key Laboratory, School of Basic Medical Sciences, Nanjing Medical University, Nanjing, 211166 China; 4 Changzhou Second People’s Hospital, Changzhou Medical Center, Nanjing Medical University, Changzhou, 213000 China

**Keywords:** Mitochondria, Brain, Neurological disorders, Mitochondrial transfer

## Abstract

Mitochondria have multiple functions such as supplying energy, regulating the redox status, and producing proteins encoded by an independent genome. They are closely related to the physiology and pathology of many organs and tissues, among which the brain is particularly prominent. The brain demands 20% of the resting metabolic rate and holds highly active mitochondrial activities. Considerable research shows that mitochondria are closely related to brain function, while mitochondrial defects induce or exacerbate pathology in the brain. In this review, we provide comprehensive research advances of mitochondrial biology involved in brain functions, as well as the mitochondria-dependent cellular events in brain physiology and pathology. Furthermore, various perspectives are explored to better identify the mitochondrial roles in neurological diseases and the neurophenotypes of mitochondrial diseases. Finally, mitochondrial therapies are discussed. Mitochondrial-targeting therapeutics are showing great potentials in the treatment of brain diseases.

## Introduction

The brain operates through a combination of electrical and chemical signals, and this process is highly energy-demanding [1, 2]. Neurons are the basic working units of the brain and are the most power-hungry cell type in the brain. The human brain contains nearly 100 billion neurons [3]. Each neuron is connected to up to 10,000 other neurons, exchanging signals via as many as 1000 trillion synapses [3]. Even in resting states or when neurons are not releasing neurotransmitters to each other, the brain consumes 20% of the body’s overall energy [4].

Mitochondria are essential for brain functions, as they produce adenosine triphosphate (ATP) to be used by brain cells [5]. Insufficient ATP supply will lead to brain cell death [6]. Reactive oxygen species (ROS) are toxic byproducts of ATP generation, produced along with the respiration process [7]. In physiological states, the ROS are maintained at a controllable steady-state level [8] and facilitate normal redox signaling of the brain cells [9, 10]. Excessive production of ROS will oxidize the brain lipids and neurotransmitters to induce enrichment of unsaturated lipids, neurotransmitter auto-oxidation, RNA oxidation, etc. [11–13]. Interestingly, mitochondria also possess antioxidant enzymes and endogenous antioxidants to balance cellular oxidation and reduction states [19, 20]. In addition, the membrane dynamics, genetic information storage and the quality control system of mitochondria are closely related to the homeostasis of the brain [14–17].

Disturbances in immune processes, protein deposition, neurogenesis, and organelle functions are reported as the pathological mechanisms underlying certain brain diseases, and all these aspects have a close relationship with mitochondrial dysfunctions. Neuronal loss in neurodegenerative diseases (NDDs) is often attributed to mitochondrial energy shortage. The neuron types affected in NDDs, such as dopaminergic neurons in Parkinson’s disease (PD) and motor neurons in amyotrophic lateral sclerosis (ALS), often have a complex structure with extensive and highly branched axonal arbors, requiring large amount of ATP for normal functions [18–21]. In healthy neurons, mitochondria provide energy for neuronal activities, and also modulate neuronal degeneration and death under stress [22–24]. Inflammatory cytokines, notably interleukin 6 (IL-6), are regarded as the endogenous biomarker and therapeutic target for depression [25]. The inflammatory cytokines can be induced by mitochondrial DNA (mtDNA) as a danger-associated molecular pattern (DAMP) via the cyclic GMP-AMP synthase (cGAS)-stimulator of interferon genes (STING) signaling pathway [26]. Epilepsy is a pathological state with abnormal electrical signals in the brain, among which the calcium signaling is a crucial point [27]. It has been demonstrated that the mitochondria-mediated calcium buffering is fundamental for neuronal activity set points in epilepsy [28, 29], which may also provide foundations for other brain pathological conditions associated with aberrant network activity. Collectively, mitochondria are involved in both the integrative mechanisms of brain diseases and specific pathologies. In this review, we summarize the roles of mitochondrial biology in brain functions and the mitochondria-dependent cellular events regarding brain pathophysiology. We also discuss the involvement of mitochondrial dysfunctions in the progression of different neurological diseases. On this foundation, mitochondrial-based therapies and advanced technologies are discussed. We highlight that mitochondrial therapy is one of the most prospecting therapeutics for brain pathology.

## Mitochondrial biology maintains brain physiology

**Fig. 1:**
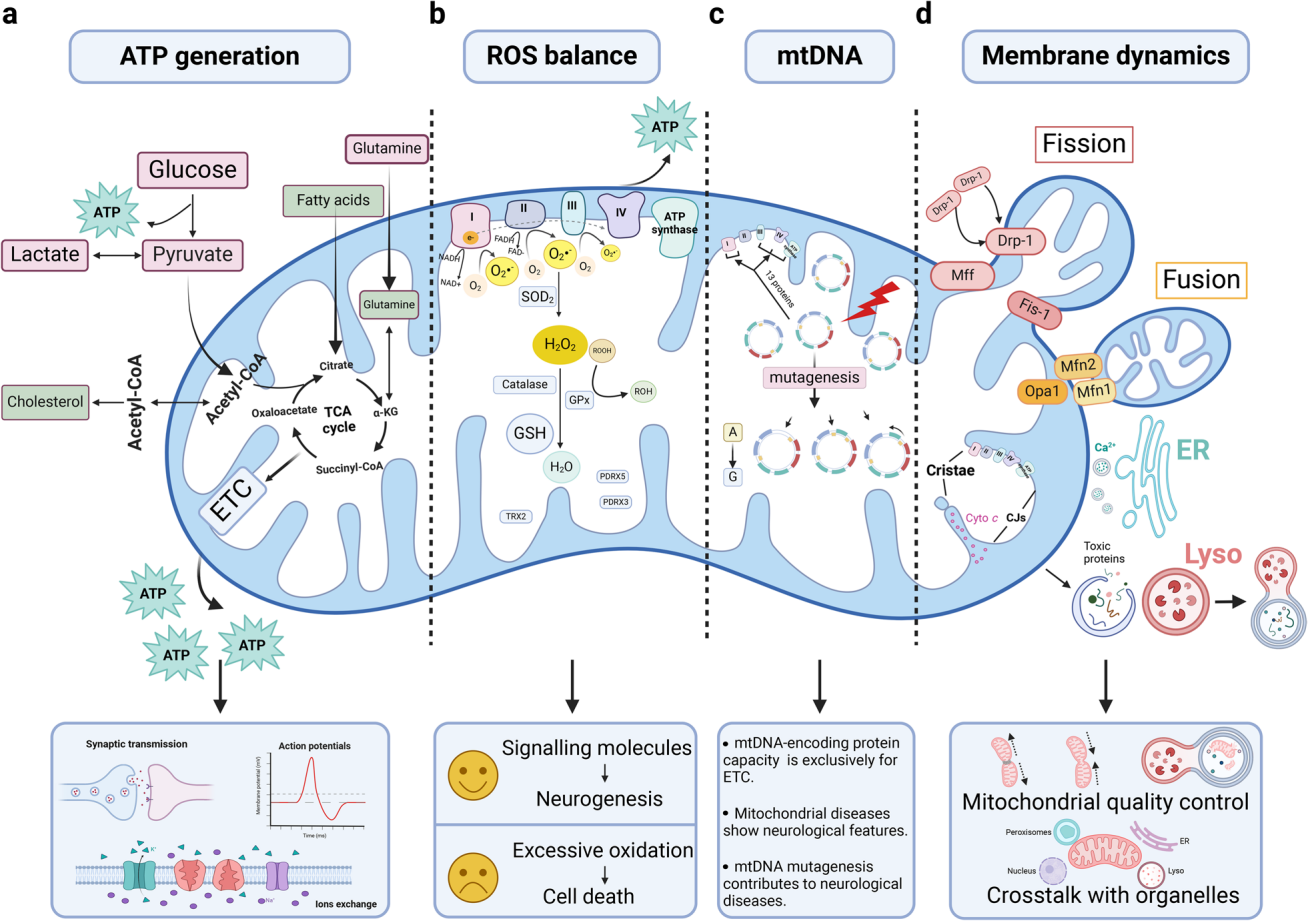
Mitochondrial biology maintains brain physiology. **a** Mitochondria are the power house and generate ATP through relevant processes of glucose, FA and amino acid metabolism. They tightly support normal brain functions dominated by neuronal activity including synaptic transmission, neuroelectrical activity, and ion exchange. **b** The mitochondrial ETC is the site of mitochondrial ROS generation. During oxidative metabolism, electrons combine prematurely with oxygen to form O_2_^•−^, which is dismutated to H_2_O_2_ by SOD2 and then converted to H_2_O by catalase and GPx. There are also mitochondria-targeted antioxidants essential for controlling ROS homeostasis in the brain, such as PDRX3, PDRX5 and TRX2. **c** The entire protein-coding capacity of mtDNA is devoted to the synthesis of mitochondrial complexes except complex II. Mutagenesis in mitochondrial genome occurs at a much higher rate than that in the nuclear genome, leading to the collapse of mitochondrial functions, which is closely related to neurological diseases. **d** Mitochondrial membrane dynamics including mitochondrial fission/fusion, membrane interactions with other organelles and ultra-structural membrane remodeling, renders the multifaceted involvement of mitochondria in cell biology. ATP, adenosine triphosphate; cyto *c*, cytochrome *c*; ER, endoplasmic reticulum; ETC, electron transport chain; FAs: fatty acids; GPx, glutathione peroxidases; GSH, glutathione; H_2_O_2_, hydrogen peroxide; lyso, lysosome; O_2_^•−^, superoxide; PDRX, peroxiredoxin; ROH, organic alcohol; ROS, reactive oxygen species; SOD2, manganese-dependent superoxide dismutase; TCA, tricarboxylic acid; TRX, thioredoxin

Mitochondria are double-membrane-bound organelles present in almost all eukaryotic cells with two aqueous compartments, the inter-membrane space (IMS) and the matrix. The IMS houses about 5% of the mitochondrial proteome [30], but is responsible for multifaceted functions including molecular exchange between mitochondrion and cytosol [30, 31], initiation of apoptotic cascades [32], biogenesis of respiratory chain complexes [33], as well as control of mitochondrial structural integrity and morphogenesis [34]. The internal matrix is the main working area of mitochondria, containing hundreds of enzymes for the oxidation of fats and carbohydrates in the tricarboxylic acid (TCA) cycle. The mitochondrial matrix and the inner membrane together constitute the functional compartment for urea cycle, protein synthesis and amino acid metabolism, supporting mitochondria as the bio-synthetic hub [35]. Beyond these metabolic substances, the mitochondrial matrix also possesses an independent genome for protein synthesis inside mitochondria [36]. In addition to the bi-layer membranes and the independent genome, another unique feature of mitochondria is their self-reproduction by binary fission, leading to mitochondrial versatile membrane dynamics, including fusion, fission, and degradation [37]. Below, we discuss the multifaceted contributions of mitochondrial biology to brain physiology (Fig. 1).

### Mitochondria feed the brain

As mentioned above, the brain has an extremely high metabolic demand. It utilizes approximately 20% of the body’s total oxygen and glucose consumption with only 2% weight [38]. About 70% of the calculated energy expenditures are used to support neuronal signaling including resting potentials, action potentials, postsynaptic receptor activation, glutamate cycling, and postsynaptic Ca^2+^ signaling, while the remainder is for non-signaling activities like biomacromolecule turnover, axonal transport, mitochondrial proton leak and actin cytoskeleton remodeling [39] (Fig. 1a). Neurons display most of the energy consumption. They generate ATP predominantly within mitochondria through oxidative phosphorylation (OXPHOS), with a small portion of ATP from aerobic glycolysis in the cytoplasm. Astrocytes are highly glycolytic and they transform glucose into lactate with low-oxygen consumption; the lactate is then delivered to neurons for complete oxidation. This process largely supports the neuronal energetic needs by supplying metabolic substrates [40, 41]. Oligodendrocytes also obtain ATP primarily by aerobic glycolysis. They use lactate for their own energy needs and also supply neighbouring axons with lactate [42]. Microglia are predominantly fueled by OXPHOS but are metabolically reprogrammed to an aerobic glycolysis-predominant phenotype under certain neurological circumstances [43, 44]. The metabolic features of the brain change with age. Throughout human lifespan, the brain glucose utilization peaks at age 4–5 years dominantly in the form of aerobic glycolysis [45]. This high-level aerobic glycolysis in the developmental stage of life is reported to support the maximal lipid biosynthesis for neurite growth [46]. Normal aging induces a global decrease in brain metabolism, with the glucose uptake decrease exceeding the oxygen use reduction, implying loss of brain aerobic glycolysis [47]. Oxidative metabolism of glucose is relatively stable, and persists to support synaptic transmission [48, 49].

It is reported that metabolic shifts in the brain contribute to neurological conditions. Disruption of mitochondrial complex I induces a Warburg-like shift in metabolism that enables neuronal survival, but triggers a progressive loss of nigrostriatal axons resembling human parkinsonism [50]. Though astrocytes possess low mitochondrial OXPHOS activity, this metabolic mode of astrocytes is indispensable for brain lipid homeostasis. Aberrant astrocytic OXPHOS would induce lipid droplet accumulation followed by development of Alzheimer’s disease (AD)-related neurodegeneration [51]. Moreover, hexokinase 2 (HK2) gates the glycolytic flux of microglia. HK2 elevation in microglia under AD and stroke pathology attenuates and promotes the pathology, respectively [44, 52], representing different effects of microglial metabolic change in brain diseases.

### Balance between ROS generation and clearance

ROS encompass a collection of molecular species derived from oxygen, including oxygen free radicals, such as superoxide anion radical (O_2_^•−^) and hydroxyl radical (•OH), and nonradical oxidants, such as hydrogen peroxide (H_2_O_2_) and singlet oxygen (^1^O_2_) [8, 53]. The major endogenous sources of ROS are trans-membrane NADPH oxidases (NOXs) and the mitochondrial electron transport chain (ETC) [8]. For ROS generated from ETC (Fig. 1b), the orderly flow of electrons down the mitochondrial ETC to complex IV results in their final deposition into oxygen to form water. However, during this process, the electrons can also react prematurely with oxygen at sites in the ETC (mainly Complexes I and III, rarely Complex II) to form O_2_^•−^, which can then be dismutated to H_2_O_2_ [8, 54]. Among NOXs, ETC and other enzymatic pathways, the mitochondrial ETC is estimated as the predominant oxidant generator in the C2C12 myoblasts [55]. Nevertheless, the assumption that mitochondria are main producers of cellular ROS is hardly conclusive [53]. The origins of cellular ROS production vary significantly, depending on the category of ROS, the types of cells, tissues or species, and the specific physiological or pathological conditions [56–58]. In the brain, mitochondria would generate more ROS under conditions of stress or gene mutations [59–62]. NOX-generated ROS are prone to be physiological second messengers and regulate the sequential and inter-dependent events of neuronal development: neurogenesis [9], neuronal polarization [63], and maturation of polarized neurons [64, 65]. Despite no unified verdict on which one is the leading ROS generator, a consensus has been reached that controlled ROS generation is critical for physiological brain function through redox-sensitive signaling pathways, while excessive ROS generation leads to oxidative stress and central nervous system (CNS) diseases [8, 66].

The mitochondria are equipped with powerful antioxidant defense systems, which are required for brain redox homeostasis (Fig. 1b). It is estimated that one-third of the cellular antioxidant enzymes (glutathione peroxidase and catalase) reside in mitochondria [53], and the manganese-dependent superoxide dismutase (Mn-SOD or SOD2) is exclusively located in mitochondria [67]. Mitochondria also contain 10%-15% of total cellular glutathione (GSH), the most abundant non-enzymatic antioxidant with a redox-active thiol [68]. There are also other antioxidants localized in mitochondria that are essential for the control of ROS homeostasis in the brain, including peroxiredoxin 3 (PDRX3), which contributes to the majority of hydrogen peroxide reduction in mitochondria and is closely related to glioblastoma therapeutics [69]; PDRX5, which protects against mitochondrial ROS and prevents hippocampal neuron death [70, 71]; and thioredoxin 2, a mitochondria-specific redox protein, homozygous stop mutation of which is reported to induce infantile-onset neurodegeneration in a 16-year-old adolescent [72].

### Independent genome, exclusive functions and mutagenesis susceptibility

mtDNA is a double-stranded circular structure (Fig. 1c). Each mitochondrion harbors 10–10,000 copies of mtDNA, in contrast to the nuclear DNA (nDNA) which contains only two copies per cell. The mitochondrial genomes are organized with factors such as mitochondrial transcription factor A (TFAM), into mtDNA-protein structures called nucleoids for gene packaging, transcription, and replication [73, 74]. mtDNA contains 37 genes, encoding 13 essential proteins of the mitochondrial ETC, as well as 22 transfer RNAs and 2 ribosomal RNAs for the function of mitochondrial ribosomes [75]. The entire protein-coding capacity of mtDNA is devoted to the mitochondrial OXPHOS [75], with the additional assistance of nuclear-encoded proteins imported into the mitochondria [76]. Of the mitochondrial ETC, the complex I is the largest component, composed of 46 sub-units with seven (ND-1, -2, -3, -4, 4L, -5 and -6) encoded by mtDNA, while the complex II genes are entirely nuclear. Meanwhile, one out of 11 sub-units of complex III (mt-CYB), three out of 13 sub-units of complex IV (mt-CO1, 2 and 3) and two of 16 sub-units of complex V (mt-ATP6, 8) are encoded by mtDNA [77, 78]. Almost all these mtDNA-encoded components form the core sub-units of complexes (except mt-ATP8 of complex V), and are conserved across all the domains of life [78].

However, mutations in the mitochondrial genome occur at a much higher rate than that in the nDNA [79, 80] due to the following reasons. First, mtDNA replication occurs continuously, depending on the cellular energy demands. The proximity of mtDNA to the sites of OXPHOS renders them more prone to be oxidized by ROS compared with its nuclear counterpart [81]. Second, limited DNA repair capacity could also be a significant factor for mtDNA mutagenesis [73]. Diseases caused by mtDNA mutation involve multiple organ systems and often present with neurological disturbances, which will be discussed in later sections.

### Membrane dynamics

Mitochondria are surrounded by a bi-layer membrane system. The outer mitochondrial membrane (OMM) serves as a platform for molecular exchange with sub-cellular compartments. The inner mitochondrial membrane (IMM) delimits the mitochondrial matrix and is further divided into the inner boundary membrane (IBM) and the cristae. The IBM hosts various channel transporters shuttling ions, ATP, ADP and small metabolites. The cristae are invaginations towards the matrix and harbor the machinery required for mitochondrial respiration [82, 83]. The mitochondrial membrane presents a high degree of morphological variability, with constant reshaping to coordinate various cellular functions (Fig. 1d). Mitochondrial fusion is the fusion of two mitochondria mediated by mitofusins (Mfn1/2) at the OMM and by optic atrophy 1 (Opa1) at the IMM [37, 84, 85]. Fission is mediated by the translocation of cytosolic dynamin-related protein 1 (Drp1) to the OMM with the guidance of fission 1 protein (Fis1) and mitochondrial fission factor (Mff) [37, 84, 86]. The two processes play important roles in maintaining functional mitochondria under metabolic or environmental stresses. Fusion could mitigate stress by mixing the contents of partially damaged mitochondria as a form of complementation, while fission enables both mitochondrial biogenesis and the removal of damaged mitochondria [87, 88].

Cristae remodeling is another aspect of mitochondrial dynamics [84, 89, 90]. The shape of cristae is constantly changing based on the metabolic state of mitochondria. The changes include increased abundance, tightening between cristae membranes, and opening of the cristae junctions (CJs, referring to the sites where crista membrane and IBM are joined) [84] (Fig. 1d). Upon respiration activation, the mitochondrion assumes a condensed appearance with the matrix contracted and cristae lumen expanded [91, 92]. Also, the cristae biogenesis increases during energy-demanding conditions [93]. Apart from involvement in OXPHOS, the CJ integrity is important for retaining the bulk of mitochondrial apoptogenic molecule cytochrome *c* (cyto *c*) inside the cristae lumen. Disruption of CJ integrity results in the egress of cyto *c* into the cytoplasm for apoptosis induction [94, 95].

It is important to note that with the membrane dynamics, the mitochondria are engaged in extensive intracellular interactions with other organelles. The mitochondria-endoplasmic reticulum (ER) contact sites are the best studied type of membrane contact, functioning in calcium signaling and lipid homeostasis [90]. ER and mitochondrial OMM form close contacts through cholesterol-rich micro-domains, called mitochondria-associated ER membranes (MAMs) [96]. The abundant lipid-synthesizing enzymes in the MAMs promote lipid synthesis, especially phosphatidylethanolamine, the main phospholipid of cell membranes [97]. Therefore, orchestrated coupling between ER and mitochondria is critical for calcium signaling and phospholipid balance.

The mitochondrial dynamics plays an important role in brain function and pathology. The fusion/fission dynamics of mitochondria is associated with the fate change of neural stem cells (NSCs) during cortical neurogenesis [28, 98]. Proteins enriched in MAMs are closely related to AD pathogenesis via regulation of lipid homeostasis [99–101]. A close link between cristae and brain has been indicated in research on Opa1. Studies have shown that reversal of Opa1-related changes ameliorates neuropathological progression by controlling mitochondrial cristae remodeling [95, 102–104]. Nevertheless, more studies on the roles of cristae remodeling and its modulatory molecules in the CNS are anticipated.

### Mitochondrial quality control

Mitochondrial quality control is a process of homeostatic regulation of the morphology, quantity, and quality of mitochondria. Besides the membrane dynamics, mitochondrial biogenesis and mitophagy are also critical aspects of quality control [105]. Mitochondrial biogenesis involves the synthesis of mtDNA, proteins, and membranes from preexisting mitochondria through mitochondrial fission [106]. Peroxisome proliferator-activated receptor (PPAR) γ coactivator 1-alpha (PGC-1α) is a key regulator of mitochondrial biogenesis [106, 107]. PGC-1α^−/−^ mice show reduced mitochondrial genes and axonal degeneration in the striatum [108], while PGC-1α over-expression increases dendritic spines and enhances the differentiation of synapses in cultured hippocampal neurons [109]. These phenotypes suggest that the PGC-1α-dependent mitochondrial biogenesis is crucial for neurite growth.

Mitophagy is a form of autophagy that selectively clears damaged mitochondria by lysosome-mediated degradation. One of the most characterized mechanisms of mitophagy is the PINK1-Parkin-mediated pathway. At basal levels, mitophagy occurs to recycle the old and damaged organelles, which may balance energy production with the demands of synaptic transmission in neurons [110]. Under proper stress, mitophagy is promoted for metabolic adjustment to external changes in metabolically enhanced neurons [111], and in differentiated oligodendrocytes [112]. However, both mitophagy defects and its broad activation may result in pathologic conditions. Mutations in PINK1 and parkin genes lead to hereditary forms of parkinsonism [113–116], while hyper-activation of mitophagy induces tauopathy-linked synaptic pathogenesis [117]. Therefore, mitophagy should be tightly controlled in the CNS.

In the brain, the mitochondrial quality control machinery shows a cell-specific pattern. Neurons are especially susceptible to mitochondrial dysfunctions for their polarized structures; therefore, recovery of stressed mitochondria or turnover of damaged ones is a critical step in the maintenance of neuronal homeostasis, especially for axons. Syntaphilin (SNPH) is a neuron-specific static anchor that immobilizes axonal mitochondria and would be released from stressed mitochondria [118]. The release of SNPH from axonal mitochondria enhances the mitochondrial retrograde transport to the soma for degradation [119]. Another regulatory machinary for mitochondrial homeostasis in axons is the m-AAA protease-dependent pathway. Mitochondrial m-AAA proteases function to remove damaged or unnecessary proteins in the IMM. Mutations in genes encoding sub-units of m-AAA protease are associated with neuronal loss and neurodegeneration in humans [120, 121]. Meanwhile, loss of the m-AAA proteases leads to mitochondrial fragmentation and deficiency in the axonal transport of mitochondria in experimental mice [122, 123]. In addition to the intrinsic mitochondrial quality control systems, neurons also maintain mitochondrial functions or dispose damaged mitochondria via communication with other cells [124, 125]. As to the astrocytes and microglia, they hold higher abilities of balancing out cellular stress via antioxidant systems, metabolic reprogramming, etc. [44, 126]. Therefore, they remain functionally healthy even after the loss of their mitochondrial functions [127], but would release signals to damage neurons [51, 128].

## Mitochondria as a multifaceted hub in brain pathophysiology

**Fig. 2:**
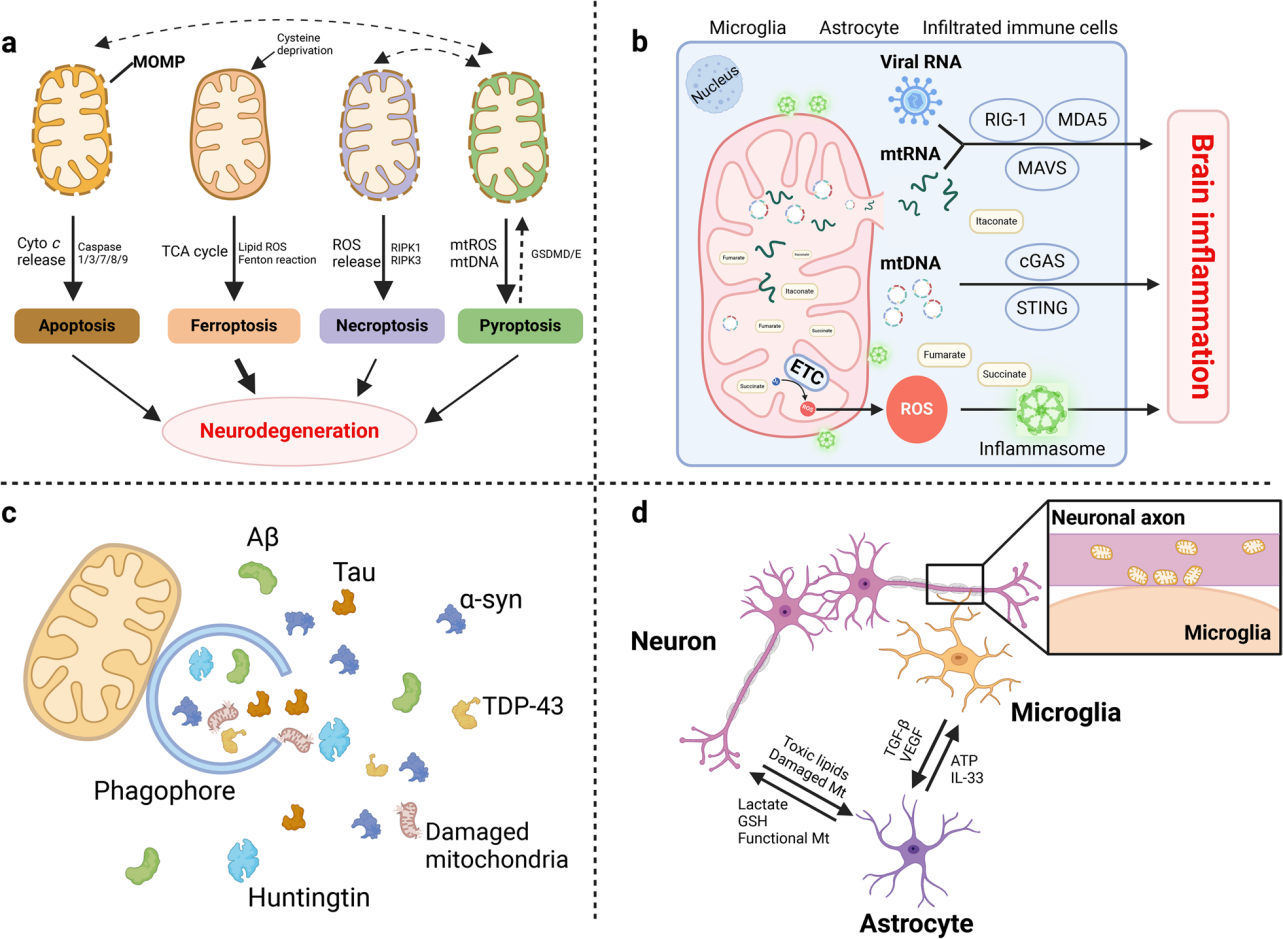
Mitochondria as a multifaceted hub of the brain pathophysiology. **a** Under cellular stresses, mitochondrial outer membrane permeabilization leads to release of cyto *c* and ROS, activating the downstream pathways of apoptosis and necroptosis. Ferroptosis is also induced by mitochondrial ETC-promotive lipid peroxide. Pyroptosis is the downstream signal of mitochondrial dysfunctions, and is controlled by mitochondria to initiate apoptosis/necrosis. **b** Mitochondria contain endogenous inflammatory inducers, including mtDNA, mtRNA, metabolic products and ROS. Mitochondria outer membrane acts as a platform for immune signaling through inflammasome and MAVS activation. MAVS also endows cells with antiviral immunity. **c** Mitochondria participate in multiple steps of autophagy including autophagy initiation, phagophore elongation, autophagic flux formation and autophagy gene induction. **d** Mitochondria participate in cellular communication in the brain through membrane contact and cellular organelle transfer. α-syn, α-synuclein; Aβ, β-amyloid; ATP, adenosine triphosphate; cyto *c*, cytochrome *c*; ETC, electron transport chain; GSH, glutathione; MAVS, mitochondrial antiviral signaling; ROS, reactive oxygen species; TDP-43: TAR DNA-binding protein 43; TGF-β, transforming growth factor β; VEGF, vascular endothelial growth factor

Mitochondria are involved in multiple signaling pathways or mechanisms, and therefore are active players in brain physiology and pathology (Fig. 2).

### Manipulators of cell fate

Mitochondria provide power for life; however, they also play roles in cell death [129] (Fig. 2a). Apoptotic cell death is a major form of regulated cell death in which two main signaling pathways are involved: the extrinsic pathway by death receptor and the intrinsic mitochondrial pathway. The mitochondrial pathway is induced by cellular stresses and requires mitochondrial outer membrane permeabilization (MOMP) to release soluble proteins (mainly cyto *c*, and also SMAC and OMI) from the IMS. The released cyto *c* initiates activation of the downstream apoptosis executioners, caspase 3 and 7, while SMAC and OMI block the caspase inhibitor to facilitate their activation [129, 130]. BCL-2 family proteins orchestrate the MOMP process and are reported to regulate neuronal death and axonal degeneration [131]. In addition to acting as a central initiator of apoptosis, mitochondria also contribute to other forms of programmed cell death [129]. Mitochondrial ROS facilitate the initiation of necroptosis by promoting the RIPK1/RIPK3-dependent signaling and necrosome formation [132, 133]. Meanwhile, mitochondrial ROS, as well as mtDNA, would evoke pyroptosis, a form of programmed cell death mediated by inflammasome and the downstream gasdermin [134–136]. During pyroptosis, mitochondrial dysfunction occurs early, and indispensably controls gasdermin D oligomerization [137] and the pore-forming activity of gasdermin D fragment for plasma membrane rupture [138]. Recently, pyroptosis has been implicated in the pathogenesis of multiple neurological diseases [24, 139–143]. Although neuronal pyroptosis has been widely studied [24, 144], glia are theoretically more likely to experience this type of inflammatory cell death with pivotal significance for disease progression. Astrocytic loss and pyroptosis-related caspase activation coexist in a mouse model of major depressive disorder [145], and inhibiting astrocytic pyroptosis alleviates depressive-like behaviors [145, 146]. Microglial pyroptosis occurs upon ALS symptom onset, and is correlated with neuronal loss [140]. Mitochondria are also the converters between the aforementioned programmed cell death events. Gasdermin E can permeabilize the mitochondria to augment apoptosis [133, 147], and pyroptotic caspase-1 is reported to initiate apoptosis and elicit secondary necrosis by activating the Bid-dependent mitochondrial apoptosis pathway [148]. Ferroptosis is a newly identified modality of regulated cell death, which is triggered by iron-dependent lipid peroxides to attack lipid membranes and tear up cells [149]. A previous study supported a critical role of mitochondria in cysteine-deprivation-induced ferroptosis through accumulation of lipid peroxides [150]. In addition, an antiviral drug for human immunodeficiency virus infection induces mtDNA stress and leads to ferroptotic cell death via autophagy [151]. Conversely, mitochondria hold the abilities to inhibit ferroptosis by mitochondrial GPx4 [152], mitochondrial dihydroorotate dehydrogenase [153], and mitochondria-localized cGAS [154]. Recently, ferroptosis has received increasing attention, especially in brain diseases [155, 156]. On one hand, iron is remarkably important in the biosynthesis of neurotransmitters, mitochondrial respiration, myelin synthesis, and among others. On the other hand, this essential micro-nutrient is engaged in catalyzing the formation of potent oxidants, exposing the nervous tissue to oxidative damage [157]. Brain iron accumulation has been found as a common feature of several NDDs [158–160]. Meanwhile, ferroptosis of neural and immune cells is linked to mitochondria and promotes neurodegeneration [161–164]. Therefore, mitochondria are considered as a central nexus between different modalities of cell death in the CNS.

### Immunoregulatory roles of mitochondria

Mitochondria lie at the heart of immunity and neuroinflammation (Fig. 2b). Expelled from stressed mitochondria, mtDNA and its oxidation products can be detected by host pattern-recognition receptors, mainly toll-like receptors, NOD-like receptors and immune interferon-stimulatory DNA receptors, to provoke inflammation via the MYD88-NF-κB signaling, the inflammasome complex and the cGAS-STING pathway, etc. [26, 165]. Meanwhile, many metabolic intermediates of mitochondria such as succinate, itaconate and fumarate are involved in inflammatory processes [166–169]. Moreover, extracellular ATP even functions as a DAMP by binding to purinergic receptors (mainly P2X7R) expressed on myeloid cells to mediate inflammatory responses, especially inflammasome activation [170, 171]. Mitochondrial ETC also participates in inflammatory events by activating inflammasomes [172]. Further, the OMM anchored by the cardiolipin acts as a platform for inflammasome localization and activation [173]. Damaged/ubiquitinated mitochondria serve as an intracellular scaffold to recruit NF-κB and activate NF-κB signaling [174]. Mitochondrial antiviral signaling (MAVS) protein endows host cells with stronger immunity against viral infection through activation of NF-κB and IRF3 to inhibit viral replication [175]. These mitochondria-related inflammatory events elucidated in the peripheral system also occur in the CNS. Abnormal protein aggregates in the ALS brain promote neuroinflammation via inducing mtDNA release and activating the cGAS-STING pathway [176]. Succinate in the cerebrospinal fluid (CSF) can be a key neuroinflammatory signal in mice with experimental autoimmune encephalomyelitis (EAE) [177]. Sphingolipid metabolism in astrocytes triggers MAVS-associated protein interaction, boosting CNS inflammation in EAE [178]. Aberrant astrocytic OXPHOS initiates brain inflammation by inducing lipid droplet accumulation [51]. It has also been reported that mitochondrial fragmentation in microglia induced by excessive injury causes irreparable neuroinflammation [128]. Suppressed bioenergetics in myeloid cells drives maladaptive pro-inflammatory responses in the ageing brain [179]. These studies collectively emphasize the involvement of mitochondria in neuroinflammation. How do these mitochondria-related immune responses further damage the neuronal cells? Cell–cell interactions may be a mechanism. Microglia with mitochondrial fragmentation release dysfunctional mitochondria to neurons and evoke neuronal damage directly [128]. Activated microglia can also induce A1 type of reactive astrocytes, which then lead to death of neurons by unknown neurotoxic substances (which might be the dysfunctional mitochondria) [128, 180]. Moreover, aberrant astrocytic OXPHOS induces lipid toxicity and astrogliosis in the brain. The lipid-overloaded astrocytes fail to provide neurotrophic and lipid-clearing support for neurons, resulting in neuronal death [51]. In addition to the abovementioned mechanisms, there may be other pathways involved in the mitochondria-related inflammatory response, which deserve explorations in the future.

### Mitochondria protect against proteinopathies by autophagy

Macroautophagy is a pathway of degradation of molecules and sub-cellular elements, including aggregates of misfolded proteins, lipid droplets, nucleic acids and defective organelles (like mitochondria, degradation of which is called mitophagy) [181]. As a lysosome-dependent catabolic process, autophagy also requires the assistance of mitochondria [182]. Specifically, newly formed membranes termed phagophores engulf the cargo, leading to the formation of double-membraned autophagosomes that get delivered to lysosomes for degradation [183]. During these processes, mitochondrial proteins interact with autophagy initiators to promote autophagy [184, 185]. Also, the mitochondrial outer membrane provides the anchorage for autophagy proteins, which is required for the elongation of phagophores [186]. Further, autophagic flux and autophagy gene induction require normal mitochondrial respiration, deficiency of which under amino-acid starvation would repress the autophagy process [187]. Additionally, ROS of the mitochondrial origin are also signaling molecules of autophagy, resulting in either survival or cell death under different circumstances [188, 189]. Mitochondrial fission and fusion could both promote autophagy in certain pathological states [190, 191]. Perturbation of the TFAM-dependent mitochondrial biogenesis induces autophagy via cytosolic mtDNA [192].

NDDs all show accumulation of abnormally folded proteins, including β-amyloid (Aβ) and Tau in AD, α-synuclein (α-syn) in PD, TAR DNA-binding protein 43 (TDP-43) in ALS and huntingtin in Huntington’s disease (HD) [193–195]. Under neurodegenerative condition, autophagic pathways are particularly important for removing unwanted proteins and damaged organelles caused by these protein deposits (Fig. 2c). It is reported that mutations in autophagy-associated genes are implicated in different NDDs. Mutations in the autophagy receptor P62 have been identified in cases of familial and sporadic ALS and frontotemporal dementia (FTD) [196]. *VPS35* is a PD-linked gene that mediates autophagosome formation and elongation, and also ensures mitochondrial stability and function [197–199]. Mutations in the autophagy gene *WDR45* cause β-propeller protein-associated neurodegeneration [200, 201]. Further, impairments in the mitochondria-dependent autophagy would decrease the proteolytic flux of α-syn and other autophagic substrates, leading to neuronal apoptosis [202]. As such, enhancers/inducers of autophagic pathways (such as latrepirdine, quercetin, trehalose and spermidine) could provide therapeutic benefit for NDDs [203–206].

### Bridge for communication between cells

Brain is a complex system of interactive networks between cells, with heterogeneous patterns of structural connections [207–210]. There are intimate communications between astrocytes, neurons and microglia, particularly the astrocyte-neuron crosstalk, in the brain (Fig. 2d). Immunofluorescence labeling showed that a single cortical astrocyte enwraps an average of four neuronal somata and up to 300–600 neuronal dendrites, and one hippocampal astrocyte contacts over 100,000 synapses [211]. These structural interactions make the basis for the formation of physiologically functional units. Special focus is given to metabolic crosstalk between astrocytes and neurons. Neurons expend a considerable amount of ATP and generate excessive ROS, while astrocytes provide neurons with metabolic substrates (lactate) and antioxidants (GSH), which are all generated from mitochondria [124, 126, 212]. In addition to energy supply, astrocytic ATP and metabolic products adenosine, glutamate and *D*-serine act on their receptors on neurons to regulate synaptic transmission, neuronal excitability and axon regeneration, establishing gliotransmitter-dependent neuron-glial networks [213–217]. It was also found that metabolic coupling of fatty acids (FAs) between neurons and astrocytes protect neurons from FA toxicity. Specifically, neurons have a low capacity for FA consumption in mitochondria for energy production. Hyperactive neurons release toxic lipids, which are taken up by neighboring astrocytes through endocytosis. These transferred FAs flow into astrocytic mitochondria for detoxification [218]. Microglia are highly sensitive to the chemical environment of the brain [219]. They identify dying cells by a wide array of signals including the release of mtDNA, ROS, apoptotic signals and metabolites to initiate phagocytosis, especially for neuronal quality control [209]. Beyond the indirect interactions between microglia and neurons though mitochondria-derived factors, microglia also monitor neuronal status via direct junctions through mitochondria-associated membranes [219].

A more straightforward interplay through mitochondria is the cell-to-cell mitochondrial transfer, in which neurons can release damaged mitochondria to adjacent astrocytes for disposal and recycling [125], and reversely, astrocytes also produce functional mitochondria to support neuronal viability [220]. Similarly, macrophages transfer mitochondria to sensory neurons to resolve inflammatory pain [221], and human brain endothelial cells transfer polarized mitochondria to neurons for protection against ischemia [222]. A general feature of this type of communication is the mitochondrial transfer into neurons for neuronal protection, which is a promising direction for developing neuron-protective methods under brain stresses. Indeed, mitochondrial transplantation is a potential therapeutic strategy. Preclinical studies delivering isolated mitochondria via intraspinal or intracerebral injection to rescue neuronal mitochondrial dysfunction have shown promising effects in acute CNS injuries and neurodegenerative disorders [223, 224].

## Mitochondria dysfunctions in neurological diseases and neurophenotypes of mitochondrial diseases

**Fig. 3:**
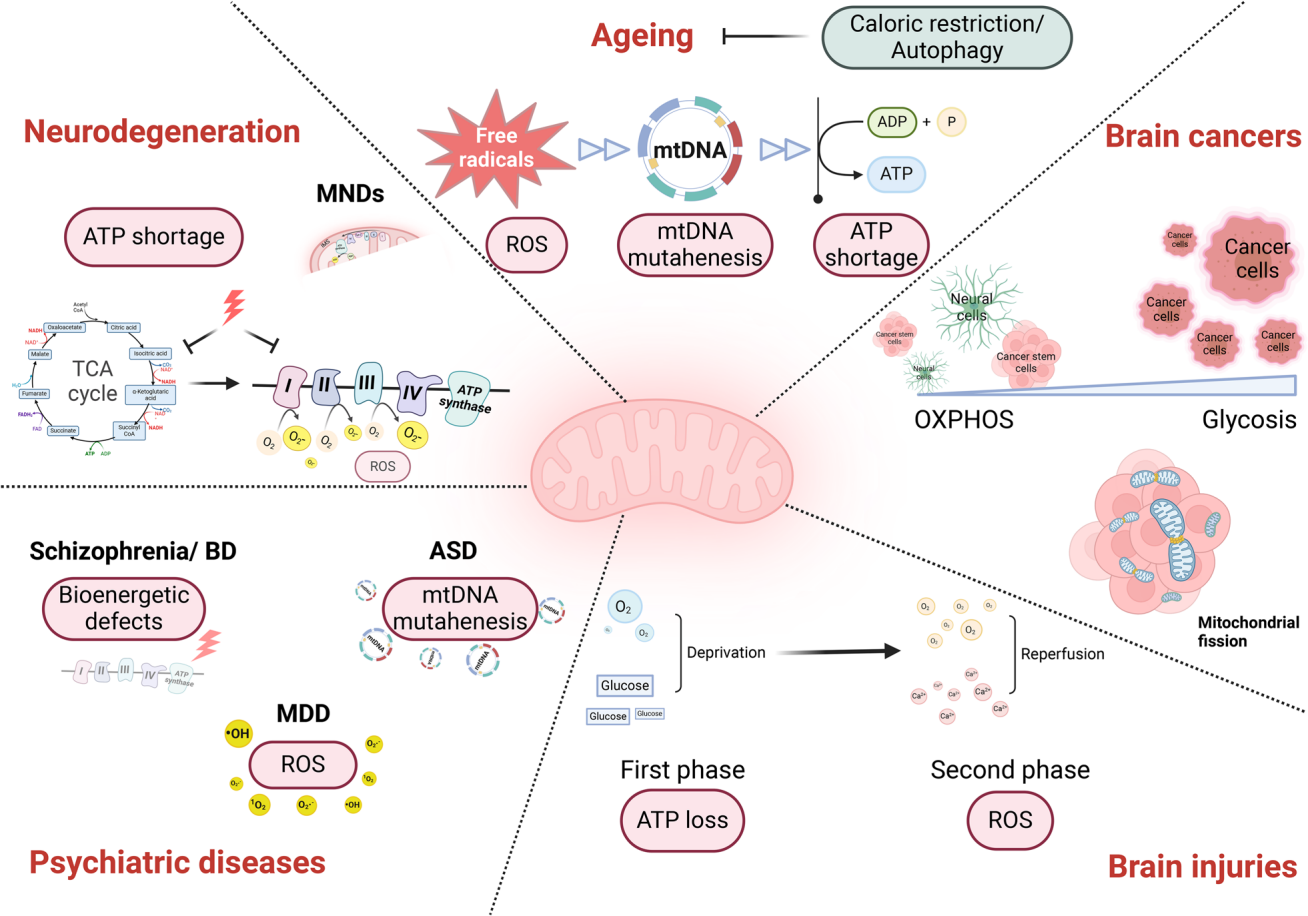
Mitochondria in neurological diseases: commonality and specificity. Mitochondrial dysfunctions are commonly seen in neurological diseases, with both commonality and disease specificity from the perspective of mechanism. ASD, autism spectrum disorder; ATP, adenosine triphosphate; BD, bipolar disorder; MDD: major depressive disorder; MNDs, motor neuron diseases; OXPHOS, oxidative phosphorylation; ROS, reactive oxygen species; TCA, tricarboxylic acid

A series of mitochondrial dysfunctions are commonly seen in neurological diseases, including energy hypo-metabolism, decreased oxidation–reduction homeostasis, decline in mitochondrial quality and activity, mitochondrial fragmentation, mtDNA damage, and pro-apoptotic activity [225–232]. In this section, we summarize recent evidence of mitochondrial dysfunction in the ageing brain and in diverse pathological conditions of the CNS including neurodegeneration, mental disorders, brain injuries, motor neuron diseases (MNDs), and brain tumors, with a particular emphasis on omics findings (Fig. 3). We also take an overview of the neurophenotypes of mitochondrial diseases.

### Ageing

Aging is a normal and inevitable process that predisposes individuals to multiple non-communicable diseases [233, 234]. Although controversy exists over whether aging falls under the category of diseases, efforts can be made to lessen its undesirable impact. Brains of aged people without a diagnosis of neurological disease are reported to show pathological changes including abnormal protein assemblies, neuronal loss and decrease of brain volume [235]. Mitochondrial impairments are also manifested in ageing brains [225]. A recent study analyzing the mammalian RNA-binding proteins (RBPs) [236] showed that Pumilio2 (Pum2) was the only transcript up-regulated in both muscle and brain samples of aged mice and humans. Multi-omics analyses further revealed that this RBP post-transcriptionally targets Mff (the mediator of mitochondrial fission) in old animals. Based on these findings, the authors suggest that modulating the Mff-linked Pum2 level/activity using genetic or pharmacological approaches may restore ageing-related mitochondrial damage, which represents a novel way for anti-aging strategy [236]. Another study presented a single-cell transcriptome atlas of the entire adult *Drosophila* melanogaster brain sampled across its lifespan, and gene network analysis revealed regulatory states related to OXPHOS [237]. Whether mitochondrial dynamics and respiration are the causes of brain ageing needs to be verified.

Starting from 1930s, researchers discovered that restriction of caloric intake increased lifespan in several species [238–241], implying that the metabolism process is important for ageing progression. Later, lifespan extension by dietary restriction is found to be mediated by the inhibition of TOR kinase [242, 243], which is a primordial negative regulator of autophagy in organisms from yeast to humans. This points to the critical role of autophagy in the ageing process. Loss-of-function mutations in proteins required for autophagy (TOR, S6K1, Sestrin1, TEFB, Beclin-1) decrease lifespan [243–247], while increased autophagy by pharmacological or genetic methods delays the overall aging and extends longevity [246, 248–252]. The pioneering Mitochondrial Free Radical Theory of Aging emphasizes that mitochondrial ROS is an important factor inducing aging [253]. However, a subsequent study showed that increased ROS production does not always shorten lifespan [254]. Subsequently, the concept that accumulation of mtDNA deletions/mutations may be important in aging came into eyes [255, 256]. mtDNA mutator mice show signs of premature aging including reduced lifespan, reduced fertility, osteoporosis, and hair graying [257]. This notion was in debate later for the observation that the increase of mtDNA mutations was actually far lower than the threshold for mitochondrial changes in normal aging mice [258]. Nevertheless, multiple issues including limitations in research methods and species differences should be taken into account in future studies [259, 260].

### Neurodegeneration

Neurodegeneration, referring to the progressive atrophy and loss of function of neurons, occurs in NDDs, including but not limited to AD, PD, ALS, HD, multiple sclerosis (MS), spinal muscular atrophy, FTD, and Creutzfeldt-Jakob disease. Ageing is the primary risk factor for most NDDs [261]. Neurons are targets of disease pathology, although selective neuronal vulnerability is shown in certain neurodegenerative circumstances [262]. Based on the high energy demand of neurons and their definite fate of demise during neurodegeneration, mitochondria appear to be a pathological mechanism of neurodegeneration [38, 262].

AD is the most common NDD, with pathological hallmarks of Aβ plaques and Tau neurofibrillary tangles in the brains of patients. Researchers from St. Jude Children’s Research Hospital profiled the whole proteome and phosphoproteome in frontal cortical samples and CSF of AD patients at different disease stages using deep multi-layer brain proteomics, and found a notable decrease of mitochondrial function in AD [263]. In their later study, further ultra-deep analysis integrating proteomes in cortex, CSF and serum of AD samples revealed that over a half of the differential proteins across three kinds of samples were strikingly mitochondrial proteins and these proteins all showed evident reduction in AD [264]. In contrast to their former study, the current ultra-deep proteomic setting detected larger numbers of differential mitochondrial proteins, which covered multiple aspects of mitochondrial function, especially metabolic processes [264]. Another study characterized tau interactomes and established that tau interacted extensively with proteins involved in mitochondrial bioenergetics. The interaction was beneficial for neuronal bioenergetics, but would decrease with disease severity [265]. Meanwhile, differential gene-expression analyses of the RNA sequencing data in MayoRNAseq dataset also highlighted the down-regulation of mitochondrial respiration and metabolism in AD [266]. A recent omics study also reported reduced mtDNA copy number and mutations of mtDNA in AD [267]. Collectively, mitochondrial energy metabolism is impaired in AD, which is also the leading player in AD progression.

Impairments in energetic pathways also occur in PD. In a study combing omics, biochemical and imaging approaches to reveal the spatiotemporal events associated with Lewy body (LB) formation, researchers found that mitochondrial respiration breakdown and reduced mitochondrial membrane potential are manifested once the LB-like inclusions are formed from α-syn fibrils [268]. Meanwhile, a quantitative proteomics study revealed mitochondrial energetic failure as the earliest event in striatal dopaminergic synapses after α-syn over-expression [269]. In summary, in NDDs represented by AD and PD, collapse in mitochondrial energy metabolism is the earliest and also principal abnormality. From the perspective of protein aggregation in NDDs, autophagy is particularly significant. Impaired autophagy is manifested in most NDDs [270–272], while autophagy enhancers counteract neurodegeneration [203, 273].

### Psychiatric diseases

The common types of psychiatric disorders include schizophrenia, major depressive disorder (MDD), bipolar disorder (BD), autism spectrum disorder (ASD), addictive behaviors, and obsessive-compulsive disorder. While the majority of patients with mental disorders appear to have widespread mitochondrial dysfunctions, the findings in patients diagnosed with certain psychiatric diseases are less consistent. There are multiple manifestations and mechanisms of mitochondrial dysfunctions in neuropsychiatric disorders, with even phasic characteristics [274–277]. Defects in bioenergetics in schizophrenia and BD are not only supported by neuroimaging methodologies [278–280], but also confirmed by transcriptomics and proteomics in body fluid samples or postmortem brains from patients [281–284]. However, transcriptomic profiling of the dorso-lateral prefrontal cortex revealed dissimilar mitochondrial alterations between schizophrenia and BD, with 41% of mitochondrial-related genes showing differential expression in the schizophrenia group, whereas 8% in the BD group [285]. This might be caused by the biphasic episodes of this neuropsychiatric disease, as mitochondrial energy metabolism in mania and depressive phases shows opposite and countervailing energy phenotypes [286–288]. ASD is highly genetically influenced, with approximately 50% heritability [289]. Whole-genome sequencing (WGS) of ASD revealed that the mtDNA variants accounted for 2% of the ASD genetic variants architecture [290]. Another study investigated the association of mtDNA heteroplasmies (co-existence of mutated and unmutated mtDNA) and content with ASD using ultra-deep sequencing, and showed that they can be used for early risk assessment of future ASD risk in newborns [277]. Although the blood-based mitochondrial respiratory chain function is not a good biomarker for MDD against controls [291], the neuron-derived extracellular vesicles in plasma of MDD subjects contain abnormal levels of proteins involved in mitochondrial dynamics, energy generation, metabolic regulation and anti-oxidant gene responses [292]. In addition, transcriptional profiling of mitochondria-associated genes in post-mortem brains of MDD subjects provided further evidence of oxidative stress in the MDD patients, showing that the ROS-related genes including *UCP4* and *SOD2* are significantly changed [293]. Some other investigations also support that chronically increased ROS production is the core mechanism in MDD etiology [294–297], as the stressful life events offer an endocrine basis for the generation of ROS [294, 295].

### Brain injuries

Brain injury refers to brain damage caused by an external force, infections, certain diseases, or a lack of oxygen. It is classified into two types depending on the original cause: (1) traumatic brain injury (TBI), like concussion, and (2) non-traumatic brain injury, like stroke, encephalitis and meningitis. The initial stages of TBI are characterized by impaired regulation of cerebral blood flow and metabolism caused by direct tissue damage and hemorrhage. The traumatic site shows an ischemic pattern with inadequate supply of oxygen and glucose, which leads to ATP depletion. The secondary injury develops over time, with release of excitatory neurotransmitters, propagation of damage through energy failure and overload of free radicals [298, 299]. Integrated spatial transcriptomes and metabolome data in injured human brain showed changes in lipid metabolism, energy metabolism, carbohydrate and amino acid metabolism, as well as antioxidant activity [300]. In the same way, stroke also faces these two mitochondrial events, the defective energy metabolism and the imbalanced redox state. An ischemic stroke event occurs when the blood flow to the brain tissue is decreased due to occluded arteries, in which lack of oxygen and nutrients leads to disturbed cellular homeostasis and, eventually cell death. During ischemia–reperfusion, oxygen is restored and ATP is replenished. However, the pro-oxidant enzymatic systems and mitochondria can also employ oxygen as a substrate to generate substantial ROS [231]. For hemorrhagic stroke, the initial bleed leads to an influx of glutamate to the brain parenchyma, which induces Ca^2+^ overload, membrane depolarization and ROS release. In the second phase of hemorrhagic stroke, ROS is generated in the way similar to the ischemia–reperfusion [301]. Encephalitis and meningitis represent the infection of the brain and the meninges caused by bacteria, virus, fungi and parasites. Mitochondrial oxidative stress underlies the cell death of regulatory T cells in an EAE mouse model [302]. Additionally, genome-wide transcriptomic analysis identified an overt reduction in mtDNA-encoded transcripts in post-mortem brain tissues of herpes simplex virus type-1 (HSV-1) encephalitis (HSE), highlighting mitochondrial damage as a critical event during HSV-1 infection [303]. Collectively, mitochondria play a role in various types of brain injuries due to its multiple functions as an energy factory, a ROS balancer, an innate immune platform, etc.

### MNDs

MNDs are a group of neurodegenerative disorders involving both the nervous system and the muscles. ALS is the most common type of MNDs, accounting for 85% of all MND cases [304]. Other types include primary lateral sclerosis, progressive bulbar palsy, progressive muscular atrophy, and spinal-bulbar muscular atrophy [305–308]. Here, we elaborate on ALS with a focus on motor neurons, to reveal the mitochondrial aspects of the MND pathogenesis. ALS is a genetically heterogeneous disorder, with landmark discoveries of the *SOD1* gene mutations in 1993 [309], and *C9orf72* in 2011 [310, 311]. As the most abundant enzyme of the SOD family, SOD1 (CuZn-SOD) is primarily a cytosolic enzyme, but it is also reported to localize within the IMS of mitochondria [312, 313]. The mitochondrial SOD1 precludes the exit of mitochondrial superoxide, and as such, protects other cell components from oxidative damage [313]. Further, targeted replacement of SOD1 only in the IMS rescues motor axonopathy of *SOD1*-deficient mice [314]. C9orf72 is found to be a mitochondria-localized protein, which can be imported into the mitochondrial IMS to regulate OXPHOS by stabilizing mitochondrial complex I assembly [315]. Dominant missense mutations in *TARDBP* gene (encoding TDP-43) can also cause ALS [316, 317], and the cytoplasmic accumulation of TDP-43 represents a pathological hallmark of ALS [318]. Compelling evidence has revealed that the disease-associated mutations of *TARDBP* increase TDP-43 mitochondrial localization and cause complex I disassembly [319], while inhibiting the mitochondrial localization of TDP-43 restores mitochondrial bioenergetic malfunctions, neuronal loss, and motor-coordinative and cognitive deficits in TDP-43^M337V^ ALS mice [320]. Apoptosis-inducing factor (AIFM1) localizes in IMS. A study has shown that patients with the Phe210Leu mutation in AIFM1 are afflicted by an inherited axonal polyneuropathy with motor axons being predominantly damaged. This disease-associated mutation in AIFM1 is sufficient to cause misassembly of mitochondrial complexes I and III [321]. Furthermore, a mitochondrial origin for ALS has also been identified in a large family with a late-onset phenotype including MND and cognitive decline, in which a missense mutation in the *CHCHD10* gene is detected. CHCHD10 is also a mitochondrial protein located in the IMS and is enriched at CJs [322]. These studies collaboratively show that gene mutations in the IMS-localized proteins lead to axonal atrophy and motor neuron degeneration, and these neurophenotypes are often associated with misassembly of the respiratory complex in mechanism. In addition to the genetic evidence for the association of MNDs with mitochondria, studies using motor neurons derived from induced pluripotent stem cells (iPSCs) from ALS and control subjects also highlighted mitochondrial genes with the most variable expression, especially those involved in the mitochondrial respiratory chain pathway [323]. Probably, motor neurons themselves determine the susceptibility of their mitochondria during MND progression. Motor neurons are highly polarized cells with an extended axon [20, 21], thus facing the special challenge of maintaining mitochondrial integrity and energy homeostasis. Neurons have an intrinsic mechanism responsible for early removal of defective mitochondria from the distal axons via the mitochondrial anchoring protein SNPH [119, 324]. Nevertheless, progressive pathological stress would deplete SNPH and compromise the SNPH-mediated regulation in later disease stages of ALS [119]. These findings all indicate a critical role of mitochondrial respiration in MND pathology, providing insights into the diagnosis and treatment of MNDs in the future.

### Brain cancers

Glioma is a common type of tumor in the brain, sometimes in the spinal cord. Among more than 120 different types of brain tumors, about 33% are gliomas. Mitochondria are involved in oncogenesis, from malignant transformation to tumor progression, and even treatment resistance, mainly through the following mechanisms: (1) metabolic flexibility via the interplay between glycolysis and OXPHOS pathways, (2) mitochondrial ROS production, and (3) functional deficits in MOMP and mitochondrial permeability transition (MPT) [325]. Preference of glycolysis over OXPHOS, enhanced ROS generation and abnormalities of mitochondria-mediated apoptotic machinery are frequently observed in various brain malignancies including gliomas [326]. In contrast to tumor cells which display glycolysis metabolic pathways, glioma stem cells rely mainly on the OXPHOS metabolic pathway [327]. Nevertheless, these brain tumor initiating cells harbor fragmented mitochondria, and inhibition of the mitochondrial fission mediator Drp1 leads to decreased oxygen consumption rate and causes metabolic stress in these cells [328].

### Coronavirus disease 2019 (COVID-19)-induced neurological manifestations

Acute infection with severe acute respiratory syndrome coronavirus 2 (SARS-CoV-2) also leads to neurological features including brain structural changes, acute encephalopathy, paralytic neuromuscular blockade, ischemic strokes and cognitive impairments [329–333]. Evidence shows that the mitochondrial mechanisms might underlie the neurological manifestations of COVID-19. The SARS-CoV-2 invading the brain can be detected in multiple brain regions with a distribution pattern consistent with neurons [329, 334]. SARS-CoV-2 enters host cells through binding of its spike protein to the receptor ACE2, which is also present on neurons [335]. Within host cells, SARS-CoV-2 hijacks double-membrane vesicles derived from mitochondrial membranes to hide and avoid attacks [336]. SARS-CoV-2 is also predicted to have a notable residency signal toward the mitochondrial matrix to alter MAVS function and mitochondrial function [337, 338]. The viral proteins/fragments would also trigger a dramatic reduction in mtDNA content in microglia, as well as activating gliosis and neuroinflammation [329, 339].

### Neurophenotypes of mitochondrial diseases

Mitochondrial diseases are caused by mutations in the nDNA and mtDNA that encode mitochondrial proteins or proteins involved in mitochondrial function. This group of diseases are multi-systemic, with substantial involvement of the nervous system. Leber’s hereditary optic neuropathy (LHON) is the first and also one of the most prevalent diseases associated with mtDNA mutations. It is an inheritable neurodegenerative disorder, mainly caused by mutations in mt-ND1, ND4 or ND6. LHON is characterized by blindness due to degeneration of retinal ganglion cells and axons of the optic nerve [340]. Besides the optic nerve, patients with LHON often show brain damage in neuroimaging [341, 342]. Kearn-Saryre-Syndrome (KSS) is caused by a large deletion of mtDNA nucleotides (ranging from 1000 to 10,000) and presents with progressive external ophthalmoplegia, atypical retinal pigmentary degeneration and heart block [343]. Neuroimaging results show cerebral and cerebellar atrophy with focal or diffuse areas of high signal intensity in certain brain regions in KSS patients [344]. Increased tau levels in the CSF are also reported in KSS [345]. In an early case report, patients with MELAS (mitochondrial encephalomyopathy, lactic acidosis, and stroke-like syndrome), mainly caused by mutations in mt-TL1, all had lactic acidosis, multiple stroke-like events with secondary neurological deficits, and radiological changes of progressive brain infarction [346]. Other mitochondrial diseases are not listed but reviewed elsewhere [347–349]. In addition to the abnormal neuroimaging results and biochemical indications, patients with mitochondrial diseases also experience cognitive problems such as memory impairment, perception deficits, and language deficits [350–352], as well as mental health problems like psychosis, chronic confusional states, hallucinations, personality change, or unsteadiness [353–355].

## Mitochondrial therapies for neurological disorders

Viable mitochondria are critical for the homeostasis of the brain, while mitochondrial malfunctions contribute to the pathogenesis of a variety of neurological conditions. Efficient clearance of damaged mitochondria through mitophagy plays a fundamental role in maintaining mitochondrial and metabolic homeostasis, energy supply, neuronal survival, and health [356]. In neurological diseases, the function of mitophagy is damaged [356], and the impaired mitochondrial functions cannot be compensated for under the condition of an irreversible injury to mitochondria [357–359]. Therefore, strategies aimed at supplementation of functional mitochondria have recently gained interest. The earliest trial was in the field of cardiovascular diseases, in which mitochondrial transplantation significantly improved postischemic functional recovery and cellular viability [360]. Later, this concept was extended to the CNS for neuroprotection and neurorecovery [220, 361–363]. Here, we summarize the rationale, application and challenges of mitochondrial transplantation for treatment of brain diseases.

### Inter-cellular mitochondrial transfer in the brain

Multiple lines of evidence suggest the presence of inter-cellular mitochondrial transfer in the brain, which not only advance our understanding of disease progression, but also provide theoretical foundations for therapeutic strategies of brain diseases [125, 363, 364]. A comprehensive summary of findings is provided in Table 1. It is reported that mitochondria of retinal ganglion cell axons are transferred to adjoining astrocytes for degradation [125]. This autophagy-assisted phagocytosis is named transmitophagy and represents a new way of mitochondrial quality control, which is predicted as a common mechanism in the nervous system [125]. Another mitochondrial quality-control process called mitocytosis has been newly launched in migrating cells like neutrophils. In mitocytosis, damaged mitochondria are released from the rear end of migrating cells to maintain mitochondrial quality and viability, which might share features with transmitophagy [365]. Although mitocytosis has not been reported in the CNS, studies are prospected for its roles in brain pathophysiology, especially within microglia of high mobility. In addition to mitochondrial quality control, mitochondria transfer participates in disease promotion. It is reported that damaged mitochondria released from microglia are sensed by astrocytes to propagate inflammatory signals and provoke neurodegeneration [128]. Nonetheless, the most important aspect of mitochondria transfer lies in its neuro-protective effects. Functional mitochondria of astrocytes are conversely transmitted to neurons, supporting neuronal viability and recovery in stroke [220], or are endocytosed by glioma cell line to inhibit malignant proliferation and enhance glioma radiosensitivity [366]. However, astrocytic mitochondria to glioblastoma can also promote a highly tumorigenic cell phenotype with increased proliferative capacity and self-renewal in a disparate transferring model [367]. Moreover, macrophages, brain endothelial cells and bone marrow mesenchymal stem cells donate their mitochondria to neurons for recovery [221, 222, 224]. Mitochondria transportation almost involves all types of cell in the brain [223, 368]. The aforementioned transferring models provide a theoretical basis for mitochondrial therapy via transplantation.

**Table 1:** Mitochondria transfer in the CNS

Donor cell	Recipient cell	Mitochondrial state	Functions	Pathological condition	References
Retinal ganglion cell	Optic nerve astrocytes	Damaged	Degrade mitochondria transcellularly	Focal axonal damage	[125]
Microglia	Astrocytes	Damaged	Evoke astrocytosis and mediate injury propagation	Neurodegeneration	[128]
Cortical astrocytes	Cortical neurons	Functional	Endogenous neuroprotective and lead to neurorecovery	Stroke	[220]
Human astrocyte cell line	Starved glioblastoma cell line	Healthy	Reactivate the mitochondrial apoptotic pathway and inhibit malignant proliferation of glioblastoma cells	Glioblastoma	[366]
Astrocytes	Glioblastoma cells	Functional	Drive proliferation and self-renewal of cancer cells	Glioblastoma	[367]
Infiltrated macrophages in the DRG	Neurons	Functional	Relieve the inflammatory pain	Chronic pain	[221]
Brain endothelial cells	Sensory neurons	Functional	Increase ATP levels	Ischemic insult	[222]
Bone marrow MSCs	Cortical neurons	Functional	Prevent neuronal apoptosis	SCI	[224]
Neural stem cells	Mononuclear phagocytes	Functional	Revert the mitochondrial dysfunction and reduce inflammatory gene in mononuclear phagocytes	MS	[368]
Human brain endothelial cell line	Brain endothelial cells	Healthy	Protect tight junction integrity of ischemic brain endothelial cells and reduce brain infarct sizes	Ischemic stroke	[370]
Human cell line	Brain cells	Healthy	Reduce neuronal disarrangement, neuronal loss and gliosis	AD	[371]
iPSC-derived astrocytes	Dopaminergic neurons	Healthy	Reverse dopaminergic neurodegeneration and axonal pruning	PD	[372]
Lymphoblasts from healthy subjects	iPSCs of schizophrenia subjects	Healthy	Support neuronal differentiation and restore functional deficits	Schizophrenia	[373]
Human umbilical cord derived MSCs	Brain cells	Healthy	Inhibit apoptosis of brain cells and decrease infarct size	Acute ischemic stroke	[374]
Hamster kidney fibroblast cell line	Cortical neurons	Healthy	Attenuate neuronal cell death and brain infarct area	Ischemic stroke	[361]
MSCs	Neural stem cells	Healthy	Prevent the loss of neural progenitor cells	Neurotoxic effects of cisplatin	[362]
Astrocytes	Cortical neurons	Functional	Protect the vulnerable neurons against toxic effects	Neural injuries after cisplatin treatment	[363]
Microglia cell line	α-syn-burdened neuronal cell line	Functional	Provide metabolic support for neurons	Neurodegeneration	[364]

α-syn, α-synuclein; AD, Alzheimer’s disease; CNS, central nervous system; DRG, dorsal root ganglia; iPSCs, induced pluripotent stem cells; MS, Multiple sclerosis; MSCs, mesenchymal stem cells; PD, Parkinson's disease; SCI, spinal cord injury

### Mitochondrial transplantation targeting brain diseases

**Fig. 4:**
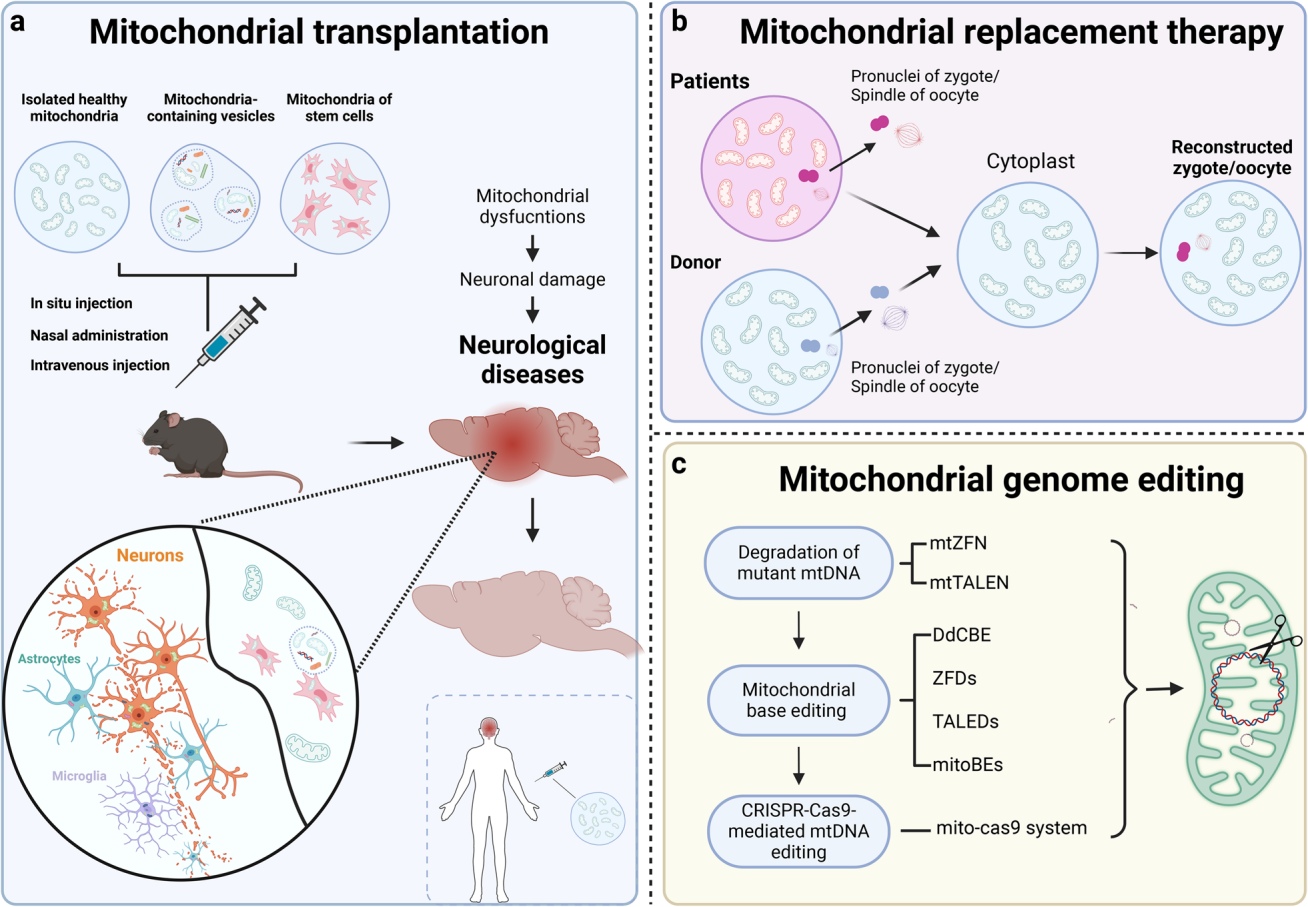
Advanced mitochondrial therapies for neurological diseases and mitochondrial diseases. **a** Mitochondrial transplantation via injection of isolated mitochondria, mitochondria-containing vesicles and mitochondria-loaded stem cells is promising for the treatment of brain diseases. **b** Mitochondrial replacement therapy is conducted by pronuclear transfer or spindle transfer. For pronuclear transfer, a zygote is generated by fertilization and then pronuclei of the zygote containing mutated mtDNA are transferred to the donor’s enucleated zygote. For spindle transfer, the spindle of the oocyte with mtDNA mutation is transferred to the donor’s enucleated oocyte, followed by fertilization. **c** Mitochondrial genome editing is conducted by editing the nuclease systems using the ZFNs, the TALENs and the CRISPR/Cas9 systems. mtTALENs and mtZFNs are mitochondria-targeted DNA nucleases and promote the degradation of mutant mtDNA for heteroplasmic shifting of mutant mtDNA. Mitochondrial base editing is achievable by DdCBEs, TALED, ZFD and mitoBEs to effectively correct the homoplasmic mtDNA mutation. The mito-Cas9 system enables successful knockin of exogenous DNA into mtDNA, which is promising for manipulating more types of mtDNA base editing. CRISPR-Cas9: clustered regularly interspaced short palindromic repeats-associated Cas9; DdCBE: bacterial cytidine deaminase fused with mitochondrial TALE-linked deaminases; mitoBEs: mtDNA base editors; MRT, mitochondrial replacement therapy; TALEN: transcription activator-like effector nuclease; ZFDs: zinc-finger deaminases; ZFNs: zinc finger nucleases

The application of mitochondrial transplantation in neurological disorders is promising (Fig. 4a). For injuries in the CNS including stroke, TBI and spinal cord injury, mitochondrial transfer has been identified as a promising therapeutic strategy [220, 224, 369]. The main strategies for mitochondrial delivery to the brain include intravenous infusion, intra-arterial injection, intraparenchymal and intracerebroventricular transplantation of isolated mitochondria, as well as intravenous delivery of mitochondria-containing extracellular vesicles. Marked effects have been observed, including inhibition of cell apoptosis and oxidative stress, reduction of infarct size and improved neurorecovery [223, 361, 369, 370]. In NDDs including AD, PD and MS, beneficial effects have been reported after intravenous, intracerebroventricular and intranasal administration as well as stereotactic injection of functional mitochondria into the targeted brain regions, or after intracerebroventricular injection of NSCs or mitochondria-containing extracellular vesicles. The beneficial effects include improved mitochondrial function, reduced neuronal loss, decreased gliosis, and significant amelioration of clinical deficits in mouse models [368, 371, 372]. For mental disorders, especially schizophrenia, injection of isolated active normal mitochondria in the prefrontal cortex prevented the occurrence of schizophrenia-like selective attention deficits in a rat model [373]. For brain malignant tumors, starvation-induced endocytosis of exogenous functional mitochondria by glioma cells inhibits their proliferation, promotes death, and enhances radiation sensitivity [366], suggesting the potential application of mitochondrial transplantation for the treatment of glioblastoma and other malignant tumors of the brain.

### Challenges and perspectives of mitochondrial transplantation

There are several challenges in mitochondrial transplantation. First, it is challenging to achieve efficient and targeted delivery of functional mitochondria to affected tissues, especially in the CNS with the existence of blood–brain barrier (BBB). Currently, the main mode of mitochondrial administration is the direct injection of isolated mitochondria into the brain lesion [361, 373]. Mesenchymal stem cells (MSCs) loaded with mitochondria also provide therapeutic support for rescuing nerve cells [362, 374], as they have been shown to cross the BBB [375]. Other optimized methods via BBB-penetrating delivery systems may also show therapeutic potentials in the future [376]. Second, retaining the activity or functionality of transferred mitochondria is also a key issue. The integrity of isolated organelles outside the cytoplasm can be fully retained only when they are separated carefully and stored in specific media [377]. In addition, peptide-mediated mitochondrial delivery has been shown as an effective method to sustain the functionality of mitochondria [378]. Third, the immune response triggered by transplanted mitochondria is also a challenge to overcome. Mitochondria derived from different sources, such as allogeneic or xenogeneic sources, may elicit immune reactions and lead to immunological rejection of the transplanted mitochondria or inflammatory responses, as is seen in mitochondrial diseases with congenital genetic mitochondrial dysfunction in all tissues. Strategies to mitigate immune responses, such as immune modulatory approaches or the use of autologous mitochondria, deserve further exploration [379]. Other challenges including ethical concerns, longitudinal effects and consequences are also of important concerns.

### Other mitochondrial therapeutic strategies for brain diseases

#### Chemicals or molecules targeting mitochondria

Some therapeutic targets or small molecules have been developed to (1) alter the mitochondrial signaling pathways or metabolic processes; (2) prevent the organelle-induced damage, including ROS, inflammation and mtDNA; (3) enhance the quality control by clearing damaged mitochondria or altering mitochondrial dynamics; and (4) induce mitochondrial biogenesis [380]. Efficacy has been shown in animal models of brain disease (Table 2).

**Table 2:** Mitochondrial therapeutics for brain diseases

Drugs/Chemicals	Mechanisms	Pathological conditions	References
*Mitochondria-targeting agents*			
MSDC-0160	Reduce the activity of mitochondrial pyruvate carrier	AD, PD	[381, 382]
NMN and NR	NAD^+^ supplement	Ageing, NDDs,	[384–387]
SBT-272	Restore mitochondrial structure and respiratory function	ALS	[388]
SS-31	Mitochondria-targeting antioxidant	AD, HD	[389, 391]
MitoQ	Mitochondria-targeting antioxidant	HD, aging-associated memory loss	[390, 391]
Trehalose	Autophagy inducer	NDDs, MNDs, MS	[203, 396–400]
Latrepirdine	Autophagy inducer	AD, PD	[204, 403–405]
Spermidine	Autophagy inducer	Ageing, neurodegeneration	[206, 250, 401, 402]
ATC161	Degradation of α-syn aggregates by p62-dependent autophagy	NDDs	[406, 407]
UMI-77	Mitophagy activator	AD	[408]
Mdivi-1	Mitochondrial fission inhibitor	NDDs, ischemic stroke	[409–411]
P110	Mitochondrial fission inhibitor	NDDs	[128]
Coniferaldehyde	Nrf2 activator that protects mitochondria by promoting mitochondrial biogenesis	AD	[420]
SBT-272	Restoring mitochondrial structure and respiratory function	ALS	[388]

Drugs	Mechanisms	Approved application	Repurposed application	References
*Repurposing mitochondria-targeted FDA-approved drugs in brain disease therapy*				
Edaravone	Mitochondria-targeting antioxidant	ALS	Ischemic stroke	[425]
Atovaquone	Inhibit mitochondrial electron transport	PCP; *Plasmodium falciparum* malaria	Toxoplasmic encephalitis	[426, 427]
Bedaquiline	Inhibit mitochondrial ATP synthase	MDR-TB	Ischemic stroke	[428]
Idebenone	Mitochondria-targeting antioxidant	LHON	AD, PD, HD	[421, 429, 430]
Pioglitazone	Agonism of PPARγ	Type 2 diabetes mellitus	AD, dementia, PD and ischemic stroke	[412–415]
Bezafibrate	Pan-PPAR activator	Hyperlipidaemia	NDDs	[418, 419]
Metformin	Induce autophagy by activation of the AMPK-mTOR signaling	Type 2 diabetes mellitus	Ageing, NDDs	[392–395]

AD: Alzheimer’s disease; ALS: Amyotrophic lateral sclerosis; LHON: Leber’s Hereditary Optic Neuropathy; Mdivi-1: mitochondrial division inhibitor 1; MDR-TB: Pulmonary multidrug resistant tuberculosis; MND: Motor neuron disease; MS: multiple sclerosis; NAD^+^ : nicotinamide adenine dinucleotide; NDD: neurodegenerative disease; NMN: nicotinamide mononucleotide; NR: nicotinamide riboside; PCP: *Pneumocystis jirovecii* pneumonia; PD: Parkinson's disease; PPARγ: peroxisome proliferator-activated receptor-gamma

Pharmacological inhibition of mitochondrial pyruvate carrier by MSDC-0160 ameliorated cerebral glucose metabolism and reduced brain damage in AD patients in a phase 2 clinical trial [381]. Experiments in pre-clinical experimental models of PD showed that MSDC-0160 exerts its effects by targeting energy metabolism [382]. Nicotinamide adenine dinucleotide (NAD^+^) is a coenzyme/cosubstrate involved in energy metabolism and energy production via participation in pyruvate dehydrogenase, TCA cycle and OXPHOS and activation of sirtuins to comprehensively regulate mitochondrial function [383]. Replenishing the NAD^+^ pool with molecules such as nicotinamide mononucleotide and nicotinamide riboside shows preventive and therapeutic effects in age-related pathophysiology and disease conditions [384–387]. SBT-272, a novel molecule targeting the cardiolipin-rich IMM for normal mitochondrial structure, functions to restore mitochondrial structure and respiratory function in motor neurons of the ALS motor cortex [388]. Mitochondria-targeting antioxidants such as MitoQ, and antioxidant peptides like Bendavia (SS31) are protective against mitochondrial damage in brain diseases [389–391]. Autophagy is a vital mechanism underlying the effects of metformin to reverse ageing and multiple ageing-related diseases [392–395]. The natural compound trehalose promotes autophagy to ameliorate neurodegeneration, MNDs and MS [203, 396–400]. Spermidine is an autophagy inducer that extends longevity [250], and ameliorates disease progression in ALS, AD and MS mouse models [206, 401, 402]. Latrepirdine improves neuropathology of AD and PD by stimulating autophagy to reduce neurodegeneration-related protein aggregates in animal models [204, 403–405]. Recently, an autophagy-based targeted protein degradation platform has been developed to synthesize chemicals for degrading deposited proteins [406]. By this platform, ATC161 was identified as a promising chemical to treat PD, AD, progressive supranuclear palsy, and ALS [406, 407]. Moreover, UMI-77 induces myeloid leukemia 1-dependent degradation of damaged mitochondria (by mitophagy) and effectively reverses molecular and behavioral phenotypes of AD [408]. Mitochondrial division inhibitor 1 mediates the repression of mitochondrial fragmentation by interfering with the Drp1 assembly at OMM and has shown clinical potential for the treatment of various NDDs and ischemic stroke [409–411]. Drp1-derived peptide, P110, has also shown neuroprotective effects in murine models of PD, AD and ALS [128]. PGC-1α and PPAR-γ activators, by stimulating mitochondrial biogenesis, show high significance in the treatment of mitochondrial dysfunction. The FDA-approved drug pioglitazone is an activator of PPAR-γ and its use is associated with a lower risk of dementia [412, 413], and lower incidence of PD and ischemic stroke [414, 415]. Bezafibrate (a pan-PPAR activator), currently used as an antilipemic agent, has been repurposed to correct metabolic defects in mitochondrial myopathies [416, 417], and NDDs [418, 419]. Coniferaldehyde, an agonist of NRF2 that protects mitochondria via targeting mitochondrial biogenesis, also attenuates AD pathology [420]. Despite the promising results in animal models, some mitochondrial therapies entering clinical trials are encountered with failure [421, 422], which may be due to the improper time points for therapy intervention. Particularly, difficulties in noninvasive assessment of mitochondrial function and damage make it hard to determine the optimal time point for the use of mitochondria-targeted compounds. Another main hurdle of mitochondria-targeting therapies for brain diseases is the insufficient brain penetration [422]. To solve this problem, nano-drug delivery systems may enable high bio-availability and targeting of specific regions, representing a promising strategy for mitochondrial drug application in neurological diseases [423]. Moreover, drug conjugation of mitochondrial-targeting moieties such as dequalinium, triphenylphosphonium, and mitochondrial penetrating peptides, can improve the entry of the drugs into the double-membraned mitochondria [424]. Additionally, approved drugs targeting mitochondria including Edaravone, Atovaquone, and Bedaquiline (Table 2), have been repurposed for the treatment of various brain diseases [425–430].

#### Mitochondrial diseases

For the mitochondrial diseases caused by pathogenic variants occurring in the nuclear or mitochondrial genome only symptomatic treatment is available by drugs, metabolic supplements and physical therapy [431–433]. Mitochondrial auto-transplantation has a rarely realistic possibility for genetic mitochondrial diseases. Mitochondrial replacement therapy (MRT) (Fig. 4b) by pronuclear transfer and maternal spindle transfer techniques has been developed in human oocytes or embryos [434, 435]. Multiple optimizations have subsequently been conducted on mitochondrial replacement technology. In 2017, Zhang et al. reported the use of MRT to treat a female case of Leigh syndrome carrying mtDNA mutation 8993 T > G. The carrier suffered from a long history of multiple pregnancy losses and offspring deaths due to this disease. She then received MRT treatment and delivered a boy with low neonatal mtDNA mutation [436]. This report raised ethical and legal controversies, as well as scientific questions, guiding the way for refinement of the techniques and highlighting the need for a robust regulatory environment and the importance of cautious clinical implementation in the future.

Genome editing techniques represented by zinc finger nuclease (ZFN) technology, transcription activator-like effector nuclease (TALEN) technology and clustered regularly interspaced short palindromic repeats-associated Cas9 (CRISPR-Cas9) technology have been comprehensively explored. Particularly, the beneficial efficacy of the CRISPR system in clinical practice has opened a new era in treating rare genetic diseases [437], and also shed light on the attempts to edit mitochondrial genome for treating mitochondrial diseases (Fig. 4c). Early studies of mitochondrial gene editing strategies are based on mitochondrial ZFN and TALEN to degrade the damaged mtDNA, which effectively shifts the heteroplasmic level of mtDNA mutation [438, 439]. For homoplasmic pathogenic mtDNA mutations, mitochondrial base editing is an effective way for single-nucleotide conversions. Later, a bacterial cytidine deaminase fused with mito-TALEN was established to induce precise manipulation of mtDNA [440]. Furthermore, other methods for mitochondrial base editing including zinc-finger deaminases [441], TALE-linked deaminases [442], and mtDNA base editors [443], provide a broader scope for mtDNA editing. Nevertheless, engineering the mammalian mtDNA by the CRISPR-Cas technology is hampered by the inability to transport nucleic acids into mitochondria [444]. Recently, a mitochondria-targeting CRISPR-Cas9 system for successful mtDNA editing has been designed, which also represents a promising approach for the treatment of mitochondrial diseases caused by pathogenic mtDNA mutations, especially those with homoplasmic mtDNA mutations [445].

## Conclusions

The mitochondrion is a complex organelle, participating in many signaling pathways and cell functions. Mitochondria are involved in the physiological and pathological processes of the brain. A deeper understanding of the basic biology of mitochondria is important for uncovering the mechanisms of brain diseases and facilitates the development of effective therapies. Despite the challenges and obstacles, therapeutic strategies of neurological diseases targeting mitochondria are worth pursuing at long last.

## Data Availability

Not applicable.

## Abbreviations

α-syn: α-synuclein
Aβ: β-amyloid
AD: Alzheimer’s disease
AIFM1: Apoptosis-inducing factor
ALS: Amyotrophic lateral sclerosis
ASD: Autism spectrum disorder
ATP: Adenosine triphosphate
BBB: Blood-brain barrier
BD: Bipolar disorder
CJs: Cristae junctions
CNS: Central nervous system
CSF: Cerebrospinal fluid
DAMP: Danger-associated molecular pattern
Drp1: Dynamin-related protein 1
EAE: Experimental autoimmune encephalomyelitis
ER: Endoplasmic reticulum
ETC: Electron transport chain
FAs: Fatty acids
Fis1: Fission 1
FTD: Frontotemporal dementia
GPx: Glutathione peroxidase
GSH: Glutathione
H_2_O_2_: Hydrogen peroxide
HD: Huntington’s disease
HSE: Herpes simplex virus type-1 encephalitis
HSV-1: Herpes simplex virus type-1
IBM: Inner boundary membrane
IFN: Immune interferon
IL-6: Interleukin 6
IMM: Inner mitochondrial membrane
IMS: Inter-membrane space
iPSC: Induced pluripotent stem cell
KSS: Kearn-Saryre-Syndrome
LB: Lewy body
LHON: Leber’s hereditary optic neuropathy
MAMs: Mitochondria-associated ER membranes
MAVS: Mitochondrial antiviral signaling
MDD: Major depressive disorder
Mff: Mitochondrial fission factor
MNDs: Motor neuron diseases
MOMP: Mitochondrial outer membrane permeabilization
MRT: Mitochondrial replacement therapy
MS: Multiple sclerosis
MSC: Mesenchymal stem cell
mtDNA: Mitochondrial DNA
NAD^+^: Nicotinamide adenine dinucleotide
NDD: Neurodegenerative disease
nDNA: Nuclear DNA
NOXs: NADPH oxidases
NSC: Neural stem cell
OMM: Outer mitochondrial membrane
Opa1: Optic atrophy gene 1
OXPHOS: Oxidative phosphorylation
PD: Parkinson's disease
PDRX: Peroxiredoxin
PGC-1α: Peroxisome proliferator-activated receptor γ coactivator 1-alpha
PPAR: Peroxisome proliferator-activated receptor
Pum2: Pumilio2
RBP: RNA-binding protein
ROS: Reactive oxygen species
SOD: Superoxide dismutase
TALEN: Transcription activator-like effector nuclease
TBI: Traumatic brain injury
TCA: Tricarboxylic acid
TDP-43: TAR DNA-binding protein 43
TFAM: Mitochondrial transcription factor A
ZFN: Zinc finger nuclease
